# Carbon Nanotubes as an Effective Opportunity for Cancer Diagnosis and Treatment

**DOI:** 10.3390/bios7010009

**Published:** 2017-02-15

**Authors:** Alessandro Sanginario, Beatrice Miccoli, Danilo Demarchi

**Affiliations:** 1 Electronics Design Laboratory (EDL), Istituto Italiano di Tecnologia, Via Melen 83b, 16152 Genova (GE), Italy; 2 Department of Electronics and Telecommunications, Politecnico di Torino, C.so Duca degli Abruzzi 24, 10129 Torino, Italy; beatrice.miccoli@polito.it (B.M.); danilo.demarchi@polito.it (D.D.)

**Keywords:** carbon nanotubes, tumor, functionalization, nanoparticle internalization

## Abstract

Despite the current progresses of modern medicine, the resistance of malignant tumors to present medical treatments points to the necessity of developing new therapeutic approaches. In recent years, numerous studies have focused their attention on the promising use of nanomaterials, like iron oxide nanowires, zinc oxide or mesoporous silica nanoparticles, for cancer and metastasis treatment with the advantage of operating directly at the bio-molecular scale. Among them, carbon nanotubes emerged as valid candidates not only for drug delivery, but also as a valuable tool in cancer imaging and physical ablation. Nevertheless, deep investigations about carbon nanotubes’ potential bio-compatibility and cytotoxicity limits should be also critically addressed. In the present review, after introducing carbon nanotubes and their promising advantages and drawbacks for fighting cancer, we want to focus on the numerous and different ways in which they can assist to reach this goal. Specifically, we report on how they can be used not only for drug delivery purposes, but also as a powerful ally to develop effective contrast agents for tumors’ medical or photodynamic imaging, to perform direct physical ablation of metastasis, as well as gene therapy.

## 1. Introduction

Current cancer treatments mostly involve surgery, chemotherapy and/or radiotherapy. The purpose of chemotherapy and of radiation is to kill the tumor cells, as they are more susceptible to the actions of these methods. However, the well-known side effects strongly minimize their actual benefits, and they reduce the potential candidates. Nanotechnology provides innovative and promising alternatives to conventional strategies to defeat tumors [1]. Among nanotechnology, Carbon NanoTubes (CNTs) have gained intensive attention and interest during the past 20 years because of their unique mechanical properties, in addition to very interesting values in electrical and thermal conductivity. Moreover, the possibility of functionalizing their surface with a wide group or bio/chemical species paves the way for numerous therapeutic and drug delivery applications [2,3,4]. For these reasons, they stand out within the newest approaches for cancer “theranostic” treatment, i.e. treatments that combine both the diagnosis and therapy in the same nanostructure.

The goal of this paper is to give an overview of the potential impact the CNTs can have on the study and treatment of cancer. In Section 2, the importance of CNTs for biosensing will be described, following in Section 3 with a specific analysis of their use for cancer diagnosis and therapy.

## 2. What Are CNTs and Why Are They Relevant for Sensing

**Figure 1:**
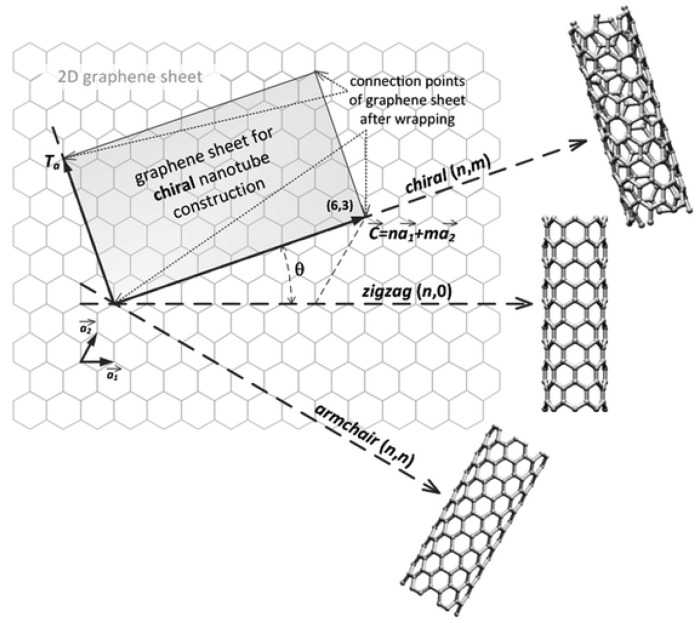
The construction of a carbon nanotube from a single graphene sheet. The chiral vector denotes different types of nanotubes. Reproduced from [9] with permission of The Royal Society of Chemistry.

CNTs are a carbon allotrope. The structure of a carbon nanotube can be seen as a single and rolled up graphene sheet. Figure 1 shows the multiple ways the graphene sheet can be wrapped. Different imaginary cut lines denote different types of CNTs with distinct properties [5,6]. Essentially, it is possible to have three cut lines, called chiral vectors, that denote three types of CNT structures: zigzag, armchair and chiral. The structure type is determined by the value of the two integer coefficients of the chiral vector, (n,m). If they are equal, the armchair structure is observed. If the two coefficients are different, the zigzag type is recognized if m = 0, else, in all other cases, we are in the presence of the chiral structure [7].

CNTs can be divided into two main groups: Single-Walled Carbon NanoTubes (SWCNTs), in which only a single graphene sheet is wrapped, or MultiWalled Carbon NanoTubes (MWCNTs), where more than one sheet is wrapped in a concentric fashion. Diameters can range from a few nanometers up to hundreds of nanometers [8].

### 2.1. Synthesis and Properties

The most used CNTs synthesis method is the low temperature Chemical Vapor Deposition (CVD) technique in which their diameter, length, purity and alignment can be easily tuned by varying process parameters (i.e., the type of precursor gas, the choice of metal catalyst, the size or roughness of the catalytic particle, the supporting substrate and any additional co-catalysts, the gas flow rate, the CVD temperature and the CVD run time). In general, CNT growth by means of CVD utilizes a carbon precursor in gaseous form fluxed with another carrier gas into a reactor chamber where a metal catalysts is needed to start the chemical reaction that leads to the growth of CNTs [10]. The two major mechanisms used to explain the catalytic growth of CNTs take their names from the position of the catalyst with respect to the substrate: tip-growth and base-growth [11]. In tip-growth mode, the active catalyst detaches from the substrate and moves forward during the growth of SWNTs [12]. In base-growth mode the whole nanotube moves away from the catalyst while the catalyst stays in its original position [13]. In both processes, the carbon precursor decomposes on the metal particle under the reaction conditions. The resulting carbon atoms, which are extremely mobile on metal surfaces, rapidly diffuse over and through the metal particles, creating a particular honeycomb tubular shape. The other two methods usually used to obtain CNTs are arc-discharge and laser ablation. The first technique consists of a DC or AC arc discharge between two carbon electrodes (usually, one of them is pure graphite) in a chamber filled with an inert gas. Many variation were applied to this method, and more details can be found in a recent review [14]. The laser ablation technique is similar to the arc-discharge with the difference that the energy is provided by a laser hitting a graphite substrate containing the catalyst material. Even in this case, there are many factors that can influence the CNTs’ properties, such as laser energy, laser peak power, laser operating mode, wavelength, target material, chamber gas and pressure [15,16].

The exceptional physical properties of CNTs are widely known in the literature [7,17]. Mahar et al. carefully reviewed different electrical, mechanical and chemical properties highly relevant for sensing [7]. Moreover, considering semiconducting nanotubes, the lower their diameter, the higher will be the energy gap [6]. This allows the synthesis of CNTs with customized electrical properties depending on the application requirements, e.g., the distinct potentials required by different molecular redox reactions. More than that, CNTs exhibit also extraordinary mechanical properties, both in terms of tensile strength and of elasticity, making them optimal candidates for the development of high sensitivity strain sensors, as underlined in detail in Mahar et al.’s review. In addition, CNT-based chemical and biological sensors have generated considerable recent research interest, as well. This is not only due to the increase of the sensor detection efficiency when CNTs are present, but also to the numerous ways in which they can be functionalized, thus enormously widening the range of potential bio-chemical applications [18,19]. Furthermore, not only the electrochemical reactivity of several fundamental biomolecules is exceptionally increased by CNTs, but also the electron-transfer reaction of proteins [20,21].

A comprehensive literature survey about electrochemical biosensors based on CNTs was recently proposed by Lawal [20]. The author reports about biosensors adopting CNTs for the detection of numerous biomolecules, like glucose, hemoglobulin, DNA, dopamine, cholesterol and others.

Recently, Wang et al. investigated the use of CNTs, and other carbon-based materials, for the fabrication of electrochemical aptasensors, i.e., sensors whose transduction is performed by aptamers [18]. Their study clearly pointed out the advantages of using CNTs for this type of sensing, which relies both on the excellent adsorption of biomolecules on the functionalized nanotubes surface and the acceleration of the electronic conversion.

However, all of the reported advantages can be useless without considering the bio-compatibility of the devices, and this aspect is detailed in Section 2.2.

### 2.2. CNTs’ Biocompatibility

CNTs seem to posses fascinating abilities of crossing the current limits, not only regarding biosensing, but nanomedicine, as well. In the last few years, more and more studies were presented in the literature about the use of CNTs for medical purposes, as will be discussed in detail in the next sections. Although the use of nanostructures in medicine (intended as objects with a diameter less than 100 nm) is promising, their potential toxicity must be also carefully assessed. Sure enough, these nanoparticles or nanostructures could not only physically cross the lung barriers, but phagocytes could in principle not be able to eliminate them, thus leading to acute inflammation [22]. Moreover, the toxic effect of CNTs can be enhanced as well by the presence of some impurities, like amorphous carbon or metallic nanoparticles, the residuals of the synthesis process [3].

Focusing specifically on CNTs, their poor solubility in an aqueous medium strongly impedes their use in almost all biological mediums [3,18,20]. It is precisely to overcome this issue that the surface functionalization of CNTs has attracted widespread interest in the last few years. The surface functionalization not only overcomes the problem of the hydrophobic nature of CNTs that tend to form very toxic aggregates [23], but primarily, it contributes to the modification of the biocompatibility of the material itself [3,24]. Mehra et al. reported about different types of functionalization of interest for biosensing applications [25]. This study addresses not only the diverse methods through which the surface can be modified (covalent or non-covalent bonding, oxidation reactions, etc.), but explores numerous biological entities that can be used to achieve this goal, as well, e.g., biomolecules, aptamers, peptides or antibodies. Eventually, they further reviewed the functionalization-dependent CNTs’ biodistribution profile, reporting that, for example, surfactant-conjugated CNTs tend to accumulate mainly in liver and spleen.

**Figure 2:**
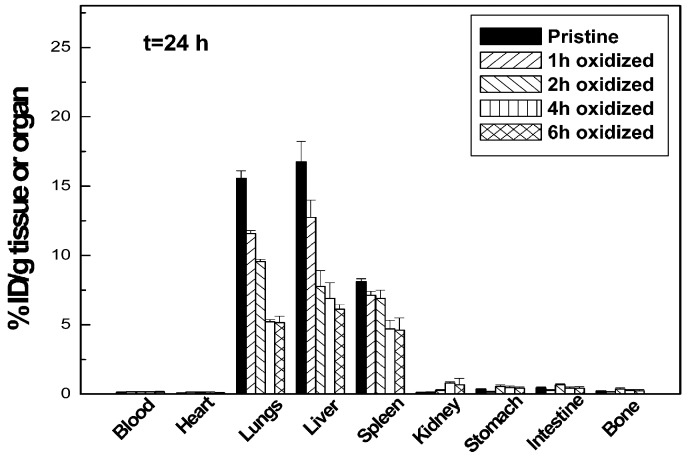
Biodistribution of multiwall CNTs in mice after 24 h of intravenous injection. Different functionalization densities are reported. Adapted with permission from [26]. Copyright 2011 American Chemical Society.

According to Jain et al., the density of functionalization is one of the key factors that impacts the CNTs’ toxicity [26]. In their study, they focused on acid-oxidized multi-walled CNTs, exploring how the degree of functionalization changes the nanotubes’ toxicity and biodistribution. As detailed in Figure 2, they observed that the toxicity of CNTs with acid-oxidized functionalization distinctively decreases if compared with the one of pristine nanotubes, suggesting that a higher toxicity is related to a lower functionalization density. Moreover, they demonstrated that the biodistribution of CNTs in organs such as liver, spleen and lungs is affected by the functionalization density.

Another key factor that affects the CNTs’ toxicity is their length. Just like asbestos fibers, non-functionalized MWNTs longer than 20 μm were found to trigger an inflammatory response and result in granuloma formation [27]. Ali-Boucetta’s work confirmed that such an inflammatory response and granuloma formation associated with CNTs depends also on their length, and that can be reduced by decreasing their effective length as a result of chemical functionalization [28].

The necessity to disarm CNTs after their use against the tumor is clear. This can be accomplished either by clearing the body of them or by enhancing their degradation.

The issue of CNTs’ biodistribution in the body, and of their excretion, was studied by many groups [29]. In particular, Lacerda et al. focused on diethylenetriaminepentaacetic functionalized single- and multi-walled CNTs further radiolabeled using ^111^indium ([^111^In] DTPA-CNTs) [3]. Their study recorded that both single- and multi-walled CNTs were eliminated from the body tissues reporting a maximum blood circulation half-life equal to 3.5 h. The CNTs’ elimination was supposed to happen through the urine. They compared their results with the ones published by Wang et al. that found 100% CNT elimination (94% excreted through the urine and 6% through the feces) using hydroxylated single-walled CNTs labeled with iodine (^125^I-SWCNT-OH) [30].

Actually, there is another very promising path to overcome CNTs’ toxicity. Up to recent years, the concept of the non-biodegradability of CNTs was widely accepted. Surprisingly, it has been proposed for the first time by Allen’s group [31] and then confirmed by other studies [32,33] that functionalized carbon nanotubes can be degraded by oxidative enzymes. Starting from this concept, Bianco’s group started working on that to design intentionally-biodegradable CNTs. In fact, they demonstrated how functionalized SWCNTs carrying defect sites on the sidewall and tips of the carbon nanotubes could be degraded by plant-derived horse radish peroxidase (HRP) [34]. CNTs are found to be biodegradable also by means of other kind of peroxidases [35,36,37], by bacteria [38] or by other biological mechanisms [39,40].

The cited works in the present section are just a few, if compared to the number of biodistribution and toxicity profiles present in the literature. Nevertheless, they provide compelling evidence of the potential future use of functionalized CNTs in nanomedicine thanks both to their limited or highly reduced toxicity and to their almost complete elimination. Nevertheless, the toxicity and the biodistribution of CNTs are strongly influenced by the physical and chemical characteristics of the final obtained nanostructure, i.e., functionalization, size, shape, etc. For this reason, it is essential to carry out individual and functionalization-specific biocompatibility studies on each new engineered CNT structure.

Because of all of the described characteristics, CNTs can be an effective solution for cancer detection, monitoring and treatment, as is analyzed in detail in Section 3.

## 3. CNTs and Cancer

The unconventional resistance of cancer cells to common anticancer treatments (i.e., chemotherapy, radiation and surgery), as well as adverse responses to these treatments are the key factors for the constantly growing research interest in cancer nanomedicine of the past years. If properly engineered, a large variety of nanomaterials can act as chemotherapeutic drug carriers, together with the ability to selectively tackle cancer tissues thanks to cutting-edge functionalizations [4]. Furthermore, recent studies are now focusing on nanoparticles combination therapy, i.e. the multi-drug delivery performed by nanoparticles able to carry and deliver different types of drugs at the same time, as extensively reviewed by [41].

The distinctive feature of nanoparticles against cancer resides in their ability to penetrate into tumor tissues, due to their dimensions, without being able to go out of cancer cells thanks to their ineffective lymphatic system [1]. This property is called Enhanced Permeability and Retention (EPR), and it underlines the motivation why nanoparticles’ dimension must be precisely tuned and designed [1].

As discussed by Lim et al., CNTs are especially suitable for such applications thanks to their hollow interior, as well as their exceptional physical properties [4]. Specifically, hydrophobic drugs can be loaded inside CNTs thanks to non-covalent π–π stacking. The targeted drug delivery is guaranteed by the outer surface functionalization tailored for specific cancer receptors. The targeted action is crucial to avoid healthy tissues’ damage, hence being less invasive for the patient.

Nevertheless, in the cancer fight, CNTs can do more than just drug delivery. Engineered CNTs are able to act as excellent adjuvant Contrast Agents (CA) for many different imaging techniques [42] and to enhance drug cytotoxicity [43]. Moreover, they can be used to perform thermal ablation [44], as well as to detect Reactive Oxygen Species (ROS) [45] or specific antigens, likewise tumor markers [46]. All these properties can be combined together to create a multifunctional tool by means of multiple functionalizations as thoroughly described in [47]. Basically, two kinds of functionalizations can be done on CNTs: covalent or non-covalent. Both of them have pros and cons, strong binding, but changed electrical properties for the covalent one and preserved electrical properties, but very weak binding for non-covalent one. For these reasons, the right functionalization strategy has to be chosen depending on the specific application [48,49].

The synergy of all of the previously-mentioned anticancer properties of CNTs is the focus of the following sections where the most recent literature works will be reviewed and discussed.

### 3.1. Contrast Agent in Medical Imaging

#### 3.1.1. Ultrasonography

**Figure 3:**
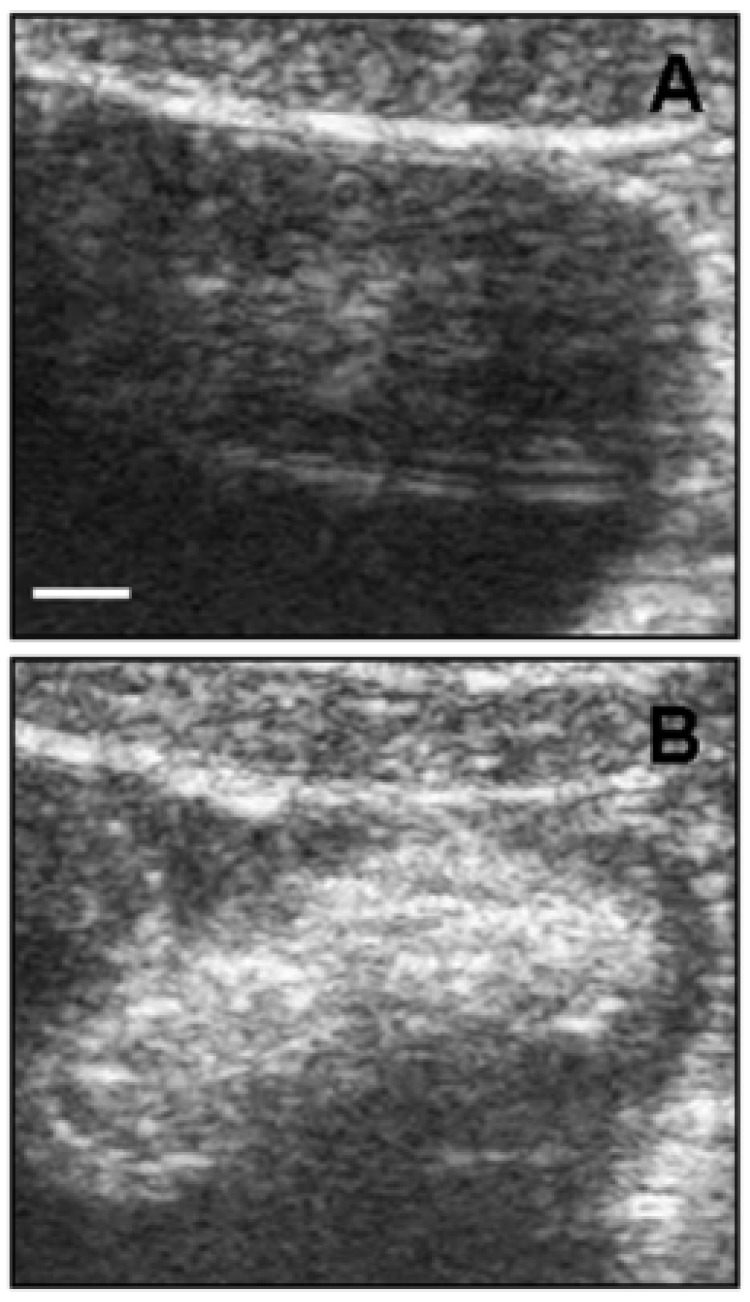
Ultrasound detection of functionalized MWCNTs in vivo. (**A**) Bladder ultrasound detection before ox-MWCNT-NH${}_{3}$${}^{+}$ injection; (**B**) ox-MWCNT-NH${}_{3}$${}^{+}$ were injected into the bladder. The images are representative results of two investigations in healthy pigs (scale bar, 5 mm). Reproduced from [52] with permission from the National Academy of Sciences of the United States of America.

Another potential and emerging powerful use of CNTs is in the field of diagnostic imaging [50]. One of the most popular imaging techniques is ultrasonography due to the low price per examination and intrinsic safety [51]. The clinical application of UltraSound (US) involves sound waves in the range of 2 to 12 MHz, providing spatial resolution in the range 0.2 to 1 mm. Delogu et al. demonstrated the superior properties as ultrasound contrast agents of functionalized MWCNTs [52]. They functionalized MWCNTs with 1,3-dipolar cycloaddition of azomethine ylides (ox-MWCNT-NH_3_^+^) and compared with other kinds of carbon nanotubes, like pristine MWCNTs and functionalized SWCNTs, showing that the functionalized MWCNTs had a signal response comparable to a commercial contrast agent. They performed the test both in vitro and in vivo on a pig showing the validity of their approach (Figure 3).

Starting from here, Wu and co-workers developed a multi-labeled MWCNT approach to target tumor cells and to exploit the MWCNTs’ properties as US contrast agent [53]. In his work, MWCNTs were covalently functionalized with PolyEthylenImine (PEI), followed by the conjugation of Fluorescein IsoThioCyanate (FITC) and PSCA monoclonal antibody (mAb_PSCA_). Such nanoprobes (CNT-PEI(FITC)-mAb) combined many functions, but the ones of interest for this review are the US contrast agent and the ability to target the desired tumor marker. In fact, in vivo results show that CNT-PEI(FITC)-mAb were able to correctly target the tumor site, leading to a clear US image of it.

#### 3.1.2. Photoacoustic Imaging

**Figure 4:**
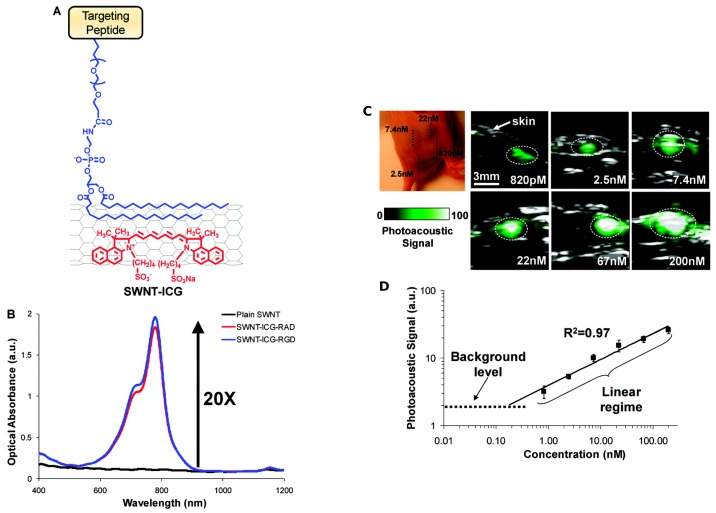
(**A**) Illustration of double CNT functionalization: IndoCyanine Green (ICG) molecules (red) and polyethylene glycol-5000 (blue) conjugated to a targeted peptide; (**B**) optical spectra of plain SWNT (black), SWNT-ICG-RGD(blue) and SWNT-ICG-RAD (red); (**C**) PhotoAcoustic (PA) detection of SWNT-ICG in living mice at different concentrations; (**D**) correlation between the functionalized CNTs concentration and the corresponding PA signal. Reprinted with permission from [60]. Copyright 2010 American Chemical Society.

Another powerful and emerging technique similar to ultrasonography is the PhotoAcoustic (PA) imaging [54]. Like ultrasonography, the PA imaging signal output is an acoustic wave, but in this case, the source is a light that induces a region of tissue to become an active acoustic source. The PA output signal is broadband. Some tissue have already an intrinsic PA behavior [54]; however, many diseases will not show PA contrast, thereby requiring the use of an exogenous contrast agent [55]. Pristine CNTs do have PA properties, and they can be easily functionalized to enhance them [56,57,58,59,60]. The problem is to target CNTs to the cancer site in order to image it. De la Zerda et al. solved this by performing a double CNT functionalization (Figure 4a). The first one was with IndoCyanine Green (ICG) dye through *π*–*π* stacking interactions, to increase the PA performances by increasing the optical absorption (Figure 4b). The second one was cyclic Arg-Gly-Asp (arginylglycylaspartic acid–RGD) peptides to PEGylated CNTs’ surface, to target the CNTs to α_V_β_3_ integrins, which are overexpressed in tumor vasculature. They were analyzed along with the control non-targeted peptide, RAD, which did not bind to α_V_β_3_ integrins.

Figure 4c shows PA images obtained by the functionalized CNTs’ contrast agent superimposed on standard ultrasound images. The authors observed a linear correlation between the functionalized CNTs’ concentration and the corresponding PA signal (Figure 4d). They calculated a 300-times improvement in sensitivity compared to plain CNTs. De la Zerda and his collaborators further developed their work by creating new PA contrast agents attaching different dyes to CNTs [61]. Another strategy to increase the PA response of CNTs was attempted by Kim et al. by covering CNTs with a gold layer known to have a very strong absorbance in the Near-InfraRed (NIR) region. The resulting structure was further functionalized with a probe that specifically recognizes the endothelium of mice lymphatic vessels. Such labeled gold-CNTs demonstrated an NIR PA response two orders of magnitude higher [62].

Wang’s group combined the two strategies together creating an RGD-conjugated silica-coated gold nanorods on the surface of carbon nanotubes for targeting gastric tumors. Results showed that the nanoprobes accumulated in the regions where the gastric cancer was present, and due to this accumulation, the PA signal was stronger over time [63].

#### 3.1.3. Near-Infrared Imaging

CNTs exhibit intrinsic fluorescence [64] and strong optical absorption in the Near-InfraRed (NIR) biological window (700 to 1400 nm). This property can be exploited as non-photobleaching fluorophores with high resolution in vivo imaging [65,66,67,68,69,70,71]. Moreover, the excitation wavelengths fall in the Near-InfraRed-I (NIR-I, 700 to 900 nm) window, while the emission wavelengths fall in the Near-InfraRed-II (NIR-II, 950 to 1400 nm) one. However, not every kind of CNT behaves in the same manner; many CNT chiralities do not have an absorption peak and, thus, are useless, if not dangerous, for such purposes. Antaris and co-workers selected only one responsive CNT chirality (6,5) and, after a proper surfactant mixing for the biocompatibility, they injected a very low amount (4 μg) of CNTs in mice [72].

**Figure 5:**
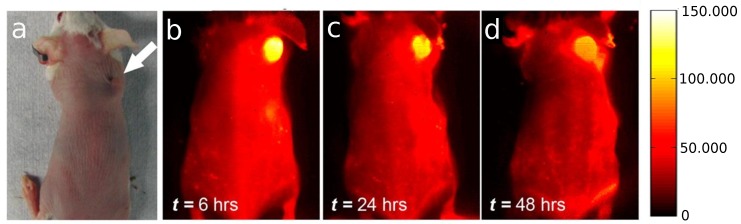
(**a**) Optical image of the tumor location in the mouse; (**b**) to (**d**) NIR-II fluorescent time course imaging 12, 24 and 48 h post-injection, from left to right, showing clear SWCNT accumulation in the tumor. The scale bar on the right corresponds to all NIR images of tumor-bearing Balb/c mice. Reproduced with permission from [72]. Copyright 2013 American Chemical Society.

Figure 5 clearly shows the accumulation and the strong NIR emission over time of CNTs injected into a mouse. The authors also used that nanomaterial to photothermally ablate the tumor, but this will be discussed in Section 3.2.

**Figure 6:**
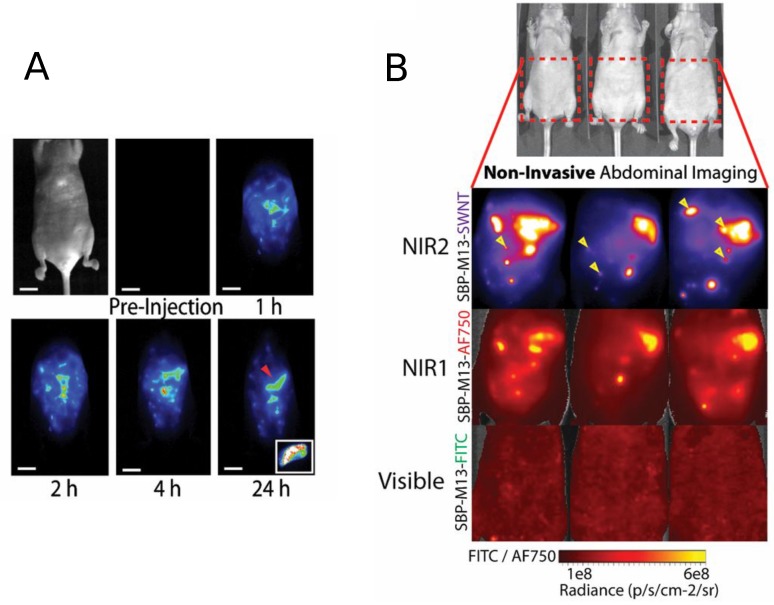
(**a**) Representative whole-abdomen NIR2 imaging series following i.p. administration of SBP-M13-SWNTs. (Inset) Surgically-excised tumor nodule (denoted by red arrow) observed 24-h post-injection of SBP-M13-SWNTs; (**b**) noninvasive imaging of ovarian tumors using SBP-M13 conjugated to SWNTs (NIR2), AlexaFluor750 (NIR1) and FITC (visible) (top to bottom). Arrows in the SWNT panel denote nodules visible only by SWNTs (*n* = 3 animals). Reproduced from [73] with permission from the National Academy of Sciences of the United States of America.

More recently, Ghosh et al. used SWCNTs as NIR imaging as a guidance for the surgical removal of small tumors [73]. Continuing their previous work [69], Ghosh and co-workers engineered M13 bacteriophage-stabilized CNTs to target the tumor and prove the photoluminescence properties in a real case. Results (Figure 6) show that very small portions of tumor become visible after a proper diffusion time. Most of them were not even visible with a standard dye. This methodology can help the surgical removal of the tumoral masses even in the early stage with a drastic increase of post-treatment survival.

#### 3.1.4. Magnetic Resonance Imaging

**Figure 7:**
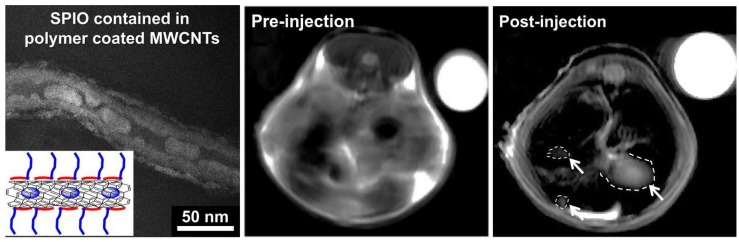
On the left: TEM image of polymer-covered SPIO-MWCNT. In vivo MRI images of mouse liver pre- and post-injection of SPIO-MWCNT (white arrows indicate tumors) compared to the internal standard (water, top right). Adapted with permission from [82]. Copyright 2005 American Chemical Society.

Magnetic Resonance Imaging (MRI) is currently one of the most powerful diagnosis tools in medical science [74]. MRI not uses ionizing radiation, but a high-intensity magnetic field to align the nuclear magnetization of hydrogen atoms in water within the body, taking advantage of the difference in water concentration among tissues to produce high-quality anatomical images with high spatial resolution [75]. MRI contrast agents are classified into two classes: spin-lattice relaxation agents, with larger effects on T_1_ shortening and spin-spin relaxation agents with larger effects on T_2_ shortening, where T_1_ and T_2_ are the proton relaxation times. In order to produce an MRI-active CNT CA, a paramagnetic or superparamagnetic agent must be conjugated to or encapsulated within the CNT platform [76,77]; moreover, a proper agent is needed to target tumors [78,79,80]. Recently, Liu et al. placed, inside the inner cavity of MWCN SuperParamagnetic Iron Oxide (SPIO), nanoparticles to create a T_2_-weighted contrast agent. To inhibit aggregation and improve their vascular biocompatibility, the SPIO-MWCNT were covered with a polymer (Figure 7, left). Diblock copolymers (PMETAC-b-PEGMA) were prepared by RAFT polymerization following the procedure described in [81]. The final polymer was composed of two units with very specific duties: the cationic PMETAC block was designed to electrostatically bind to the negatively-charged oxidized CNTs, while the second functional PEGMA block was designed to improve the aqueous dispersion stability of the hybrid material.

**Figure 8:**
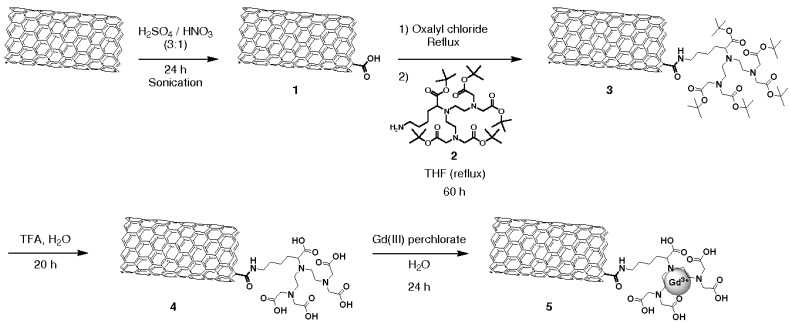
Functionalization steps to obtain Gd-CNTs. Reproduced from [89] with permission from John Wiley and Sons.

Figure 7 (middle and right) shows in vivo MRI measurements’ image pre- and post-injection. A 55% increase in tumor to liver contrast ratio was observed comparing the two images, thus enhancing the detection of the tumor [82]. One of the most studied and used CA for the T_1_ shortening is the Gd${}^{3+}$ ion [83,84,85], also in combination with CNTs [86,87]. To exploits such ions, Jahanbakhsh et al. modified the so-called gadonanotubes (i.e., gadolinium-linked carbon nanotubes) with diamine-terminated oligomeric poly(ethylene glycol) via a thermal reaction method in order to improve their water solubility and biocompatibility. The created complex increased the tumor targeting by means of a better EPR effect of tumor blood vessels [88]. Another effective way to exploit Gd${}^{3+}$ ions is the one used by Marangon’s group. They functionalized MWCNT with a molecule called diethylenetriaminepentaacetic dianhydride (DTPA); such a molecule is said to be a chelating molecule; in other words, it has the ability to strongly bond (to chelate) another molecule or ion (Gd${}^{3+}$ in this case) to itself. Bonding between MWCNT and DTPA was covalent, while the one between DTPA and Gd${}^{3+}$, despite the fact that it was not a covalent one, was still very strong, resulting (Figure 8) in a very stable compound with no risk of Gd${}^{3+}$ release over time.

In vivo MRI on mice, in which Gd-CNTs were administrated intravenously, confirmed Gd-CNTs’ uptake in liver and spleen, demonstrating the ability to act as a good CA despite their cellular internalization. In addition, such a complex can benefit both from T_2_ intrinsic contrast of oxidized CNTs and from T_1_ contrast derived from gadolinium ions, to create a clear MR image [89].

#### 3.1.5. PET/SPECT

Positron Emission Tomography (PET) is a noninvasive nuclear imaging technique, which employs a radiolabeled molecular ligand (or tracer) to track cellular activity. Among the nuclear imaging techniques, PET has become an important tool in both clinical and research fields. Two rotating detectors identify gamma (γ) rays emitted from a decaying radionuclide inside the patient and convert the detected energy into an electrical signal, which is then processed into an image. In PET imaging, radionuclei release positrons, which interact with electrons in the immediate area, and the result is the emission of two very high energy (511 keV each) photons. The two 511-keV photons are emitted in opposite directions at approximately 180 degrees from each other. The γ rays are detected by a ring of detectors that register coincident rays coming from a single event along a given line of response.

Positron-emitting radionuclides can be conjugated or even inserted into CNTs for PET imaging. Liu et al. first demonstrated tumor-targeted PET imaging with radio-labeled SWNTs functionalized with phospholipids bearing polyethylene-glycol (PEG) linked to an arginine-glycine-aspartic acid (RGD) peptide. Such a complex achieved an efficient targeting of integrin-positive tumor with a consequent good contrast imaging [90]. Similarly, other CNTs’ functionalization, both with radio-labeled and tumor targeting biomolecules, works have been explored for multimodality cancer theranostics [91,92,93].

**Figure 9:**
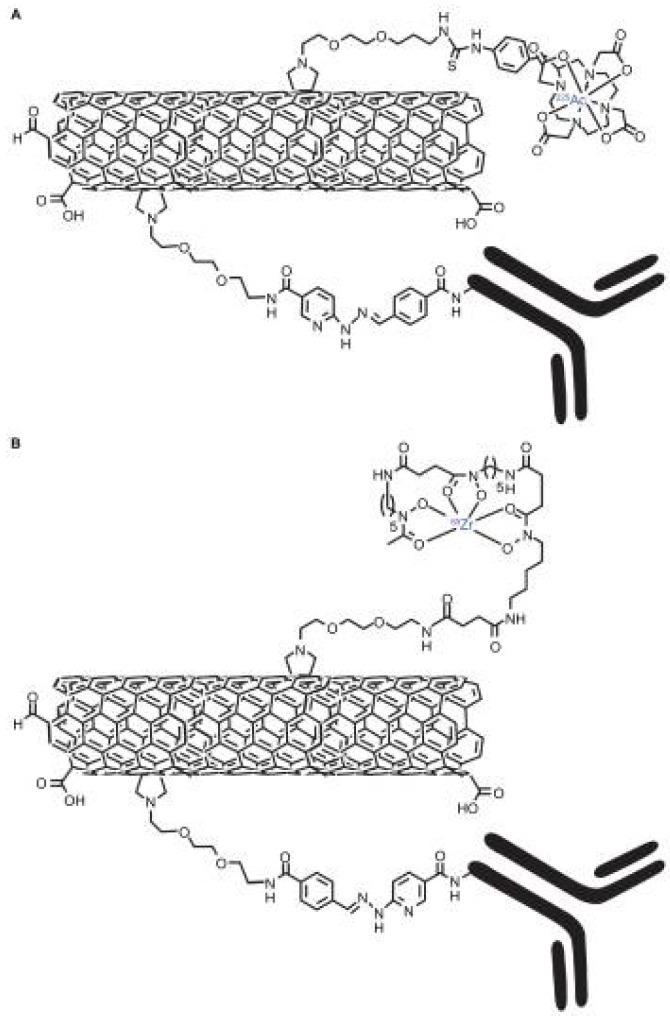
Graphical representations of the key moieties that were appended to the water-soluble SWCNT-NH2 by covalent-functionalization with radionuclides, 1,4,7,10-tetraazacyclododecane- 1,4,7,10-tetraacetic acid (DOTA), desferrioxamine B (DFO) and antibodies (note, not drawn to scale). (**A**) Radioimmunotherapeutic drug Construct I (SWCNT-([225Ac]DOTA)(E4G10)); (**B**) radioimmunoimaging drug Construct II SWCNT-([89Zr]DFO)(E4G10). Reproduced from [94]. Copyright 2010 Dove Medical Press Ltd.

Ruggiero and coworkers developed two different types of MWCNT constructs in which they added the E4G10 antibody to specifically target its epitope expressed in the tumor angiogenic vessels. The first one was designed also to deliver the potent alpha particle-emitting ${}^{2}$25Ac${}^{3+}$ radionuclide generator (Figure 9a), while the other to deliver the positron-emitting ${}^{8}$9Zr radionuclide for PET imaging (Figure 9a). Using the second construct for the PET imaging, they obtained an approximately five-fold increase in specific activity, which improved the signal-to-noise ratio of the image [94]. While obtaining excellent results for cancer therapy with the first construct, it has to be noted that a single CNT could be designed to incorporate both the imaging [95] and therapeutic cargoes [96] onto the same platform.

#### 3.1.6. Multimodality Imaging

It should be clear at this point that every imaging technique has its pros and cons, thus impeding one approach to prevail on an another. The next ideal step, then, is to combine all of the pros in a single technique avoiding all of the cons. For this reason, many research groups are working on multimodal CAs that can exploits all of the benefits deriving from each technique [47,97,98]. CNTs are an ideal platform for this duty due to the multiple types of functionalizations that are possible to perform on them [99]. For example, Cinsneros et al. created a double CA based on CNTs. They filled CNTs with Gd${}^{3+}$ and ${}^{64}$Cu${}^{2+}$, respectively, for MRI and PET systems. Despite that they did not test them on actual tumors, they proved platform stability over time in vivo and verified their accumulation mainly into lungs and liver [95]. This represents one example of the direction taken in the design of advanced imaging probes with variable multimodal imaging capabilities. In a similar way, Wang’s group designed and realized a bimodal CNT CA for SPECT and MRI. Superparamagnetic Iron Oxide Nanoparticles (SPION) granted the magnetic properties to the CNT, while ${}^{99m}$Tc granted the radioactive property. Even in this case, no actual tumor was present in the test subject, but only stability, distribution and organ accumulation were studied. Distribution studies showed prevalent accumulation in the lungs, liver and spleen with a gradual decrease in lung uptake, while the liver signals remained constant after 4 h. Imaging results demonstrate the capability of the hybrids as dual MRI and SPECT contrast agents for in vivo use [100].

Very recently, Zhao et al. created a new CNT multimodal hybrid by combining SWCNT, coated with polydopamine (PDA) and PEG, with Mn${}^{2+}$ for the MR imaging and ${}^{1}$31I for nuclear imaging, as well as radioisotope cancer therapy. As revealed by MR and gamma imaging, efficient tumor accumulation of SWNT@PDA-${}^{1}$31I-PEG was observed by the authors after systemic administration into mice. Moreover, they exploited the strong NIR absorbance of SWNTs, to perform NIR-triggered photothermal therapy in combination with ${}^{1}$31I-based radioisotope therapy to destroy the cancer with good results [101]. Such emerging multimodal contrast agents will be a valuable tool for tumor imaging, as well as theranostic and guidance applications in radiation therapy [102].

### 3.2. Photothermal Therapy

Hyperthermia is a therapeutic procedure used to increase the temperature above a cellular threshold and, thus, inducing cell death in a region of the body affected by cancer [103,104,105,106]. When the temperature is increased by means of optical radiation, it is often called PhotoThermal Therapy (PTT). The advantage of thermal ablation over surgical tissue removal is its minimally-invasive nature with reduced mortality, as well as the potential to treat tumors in vital structures that simply cannot be removed surgically. Moreover, PTT can synergically enhance tumor cytotoxicity when combined with other treatments, like chemotherapy or radiotherapy [107,108]. Hyperthermia also preferentially increases the permeability of tumor vasculature, which can enhance the delivery of drugs into tumors [109]. Reliably surpassing the cellular heat resistance threshold is the key factor for the thermal ablation of cancer cells [110]. The ability of CNTs to convert near-infrared light into heat led many research groups to create a new generation of hybrids for cancer phototherapy with high performance and efficacy [44,111,112,113,114,115].

Hashida et al. designed and realized a novel SWCNT composite with a peptide having a repeated structure of H-(-Lys-Phe-Lys-Ala-)${}_{7}$-OH ((KFKA)${}_{7}$). (KFKA)${}_{7}$ was designed to wrap SWCNTs and to have good dispersibility and stability in water. Both in vitro and in vivo tests gave good results; in fact, hybrids, directly injected into inoculated colon 26 tumor, followed by NIR irradiation rapidly increased their temperature to 43 ${}^{\circ }$C, leading to a conspicuous tumor suppression [116].

Exploiting the excellent CNTs NIR absorption is not the only way to increase their temperature [117,118]. Gannon’s group covered SWCNTs with a polymer to avoid their aggregation and injected them into tumor. After that, they exposed polymer-coated CNTs to a 13.56 MHz RF field for 2 min. They found that on all test samples, the tumors were completely destroyed. On the contrary, tumors without CNT, but under the same RF field and tumors with CNT, but without RF field, were still viable. Such results suggested that CNT hyperthermia therapy may allow noninvasive RF field treatments to produce lethal thermal injury to the malignant cells [118].

### 3.3. Photodynamic Therapy

**Figure 10:**
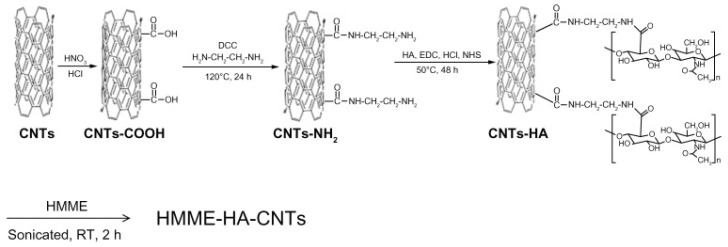
Scheme of the preparation of Hematoporphyrin MonoMethyl Ether Hyaluronic Acid Carbon Nanotubes (HMME-HA-CNTs). Reproduced from [122]. Copyright 2013 Dove Medical Press Ltd.

Small amounts of Reactive Oxygen Species (ROS) are constantly generated in organisms as a consequence of aerobic metabolism. Living cells have some countermeasures to nullify such toxic species, called antioxidants. When the ROS are too high with respect to antioxidants, there is a condition referred to as oxidative stress. When oxidative stress persists for too long, in a cell, it can lead to cell death, called apoptosis [119]. Apoptosis and cancer are opposed phenomena, but ROS have been widely reported to play a key role in both. In the scope of tumor treatment, ROS are intentionally induced inside cancer cells to kill them [120]. PhotoDynamic Therapy (PDT) is a minimally-invasive technique that exploits special PhotoSensitizers (PS) that, upon illumination, generate ROS. In this context, nanomaterials and in particular CNTs can play an important role [103,121]. Shi et al. developed a novel hybrid by functionalizing CNTs with Hyaluronic Acid (HA-CNT) making them highly dispersible in water [122]. In a second step, they adsorbed a PDT agent called Hematoporphyrin MonoMethyl Ether (HMME) on the top of HA-CNT, as depicted in Figure 10.

In vitro and in vivo tests were conducted on the samples to test their photodynamic and photothermal properties. Concerning the in vivo tests, comparison tests were conducted. The tumor-bearing mice were divided into five groups and administered differently: (1) saline solution; (2) HA-CNTs and treated with an 808-nm laser; (3) HMME and treated with a 532-nm laser; (4) HMME-HA-CNTs and treated with only a 532-nm laser; (5) HMME-HA-CNTs and treated with both 532- and 808-nm lasers.

**Figure 11:**
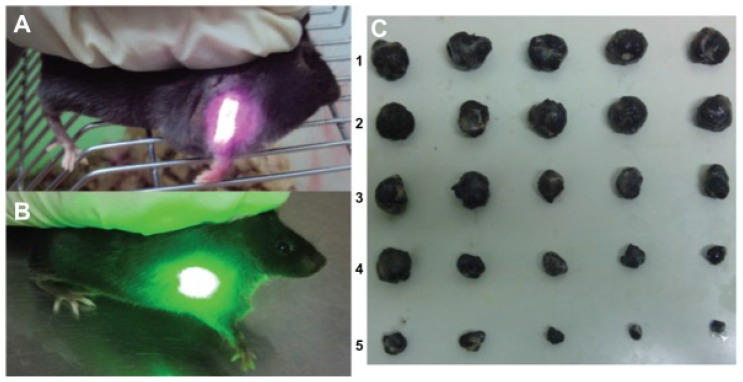
Tumor-bearing mice treated with: (**A**) an 808-nm near-infrared laser and (**B**) 532-nm laser; (**C**) photo of tumors taken out of the saline group (1), the HA-CNT (808 nm) laser group (2), the HMME (532 nm) laser group (3), the HMME-HA-CNT (532 nm) laser group (4) and the HMME-HA-CNT (532/808 nm) laser group after treatment for eight days (5). Reproduced from [122]. Copyright 2013 Dove Medical Press Ltd.

Figure 11 summarizes the obtained results. After eight days, the tumors where extracted and analyzed. As can be seen from Figure 11C, Groups 1, 2 and 3, which were the control groups, do not have a substantial reduction in dimension. On the opposite side, the group treated with PDT (Group 4) resulted in a noticeable tumor reduction and, even more important, when treated in synergy with NIR photo-thermal therapy (Group 5) [122]. There results suggests great promise for PDT [123,124], especially in combination with other therapies [125,126,127].

### 3.4. Drug Delivery Systems

The aim of chemotherapy, the method of choice in cancer treatment, is to destroy cancer cells whilst minimizing side effects to healthy tissue. However, many current chemotherapeutic agents are far from ideal. In fact, many of them have a high general toxicity. Moreover, they have additional drawbacks, like limited solubility and a poor non-selective biodistribution. To overcome these issue, scientists started to use CNTs as targeted drug delivery [128,129,130,131,132,133]. As already mentioned, CNTs exhibit properties that can be exploited for designing excellent drug nano-vectors. Some of these features are, for example: enhanced EPR effect [134], a needle-like shape that facilitates transmembrane penetration and intracellular accumulation [135,136] and the easiness of molecule loading onto the surface or within the interior core of CNTs via both covalent and non-covalent interactions [137,138,139,140].

Ji et al. non-covalently functionalized SWCNTs with chitosan by wrapping it around the CNT surface. Chitosan functionalization made CNTs dispersible in water and more biocompatible. To target the new material only to the cancer cell, Ji’s group further functionalized it with folic acid because the folate receptor is overexpressed in many tumors. Finally, they attached the well-known anti-cancer agent Doxorubicin (DOX). The final hybrid, thus, was tested in vitro and in vivo and showed better results in killing tumors cells, even at a dose much lower than free DOX. The authors ascribe these good results to three primary factors: the more precise tumor targeting due to the folic acid, the better ability of the CNTs to enter inside the cells with respect to the free DOX and the fact that the DOX release happens in a low pH environment, typical of tumors [141]. It is clear that tumor targeting increases efficacy and reduces the required drug dose [142]. Moreover, much research is going toward multifunctional CNTs, so they can serve as imaging contrast agents while they delivery therapeutics [53,143,144,145].

### 3.5. Intentional Cytotoxicity

It is now generally accepted, in the scientific community, that CNTs are able to cross the cellular membrane, independently of the cell type [1,4,134]. In this perspective, CNTs show two sides of the same coin: the intended cytotoxicity to kill cancer cells and the unwanted cytotoxicity resulting from using them for other therapies, like drug vectors, contrast agents, and so on. While we already discussed the latter one in Section 2.2, some words are worth being addressed to the first one. Many research groups are working on exploiting CNTs’ cytotoxicity for destroying cancer cells [146]. Among them, Garcia-Hevia’s group is one of the most active in this field. They tested the cytotoxicity of bare MWCNTs, and they found that they can easily enter inside the cells and intermingle with the protein nanofilaments of the cytoskeleton, interfering with the mitosis (cell division) biomechanics. Such a behavior mimics the effect of some commonly-used anti-cancer drugs, like paclitaxel [147].

### 3.6. Gene Therapy

Gene therapy can be defined in the most general sense as a method to provide a patient somatic cells with the genetic information required for producing specific therapeutic proteins to correct or modulate disease [148]. For successful gene therapy, it is necessary to develop vectors capable of efficiently introducing genetic materials into target cells with minimal toxicity. Among the various gene carriers, viral vectors are still the most widely investigated because of their efficient transfection efficiency [149]. However, the safety concerns regarding the use of virus in humans and some other limitations make non-viral delivery systems an attractive alternative [150]. Non-viral vectors are particularly suitable with respect to simplicity of use, ease of large-scale production and lack of specific immune response. Recently, several novel non-viral vectors have been developed that approach viruses with respect to transfection efficiency [151]. Among these, CNTs attracted much interest due to their ability to easily penetrate the cellular membrane and low immunogenicity [128,152,153,154,155]. For example, in a continuation of a previous study about tumor apoptosis, delayed tumor growth and prolonged survival of human lung xenograft-bearing mice after intratumoral administration of small interfering RNA (siRNA)-functionalized MWCNTs complexes [156], Guo et al. functionalized MWCNTs with a proper siRNA, whose effect is the inhibition of polo-like kinase 1 (PLK1) that is predicted to block the reproduction of cancer cells (lung cancer in this case). They compared CNTs and cationic liposomes as vectors by means of intratumoral injections. They found that siPLK1 with MWCNTs facilitated its internalization by tumor cells in solid tumor mass in vivo, resulting in significant PLK1 knockdown compared to cationic liposome complexes, even though the latter complexes are usually injected systemically [157]. In the same way, Siu’s group non-covalently functionalized CNTs with a polymer (succinated polyethylenimine) and to a siRNA fragment to silence genes of a melanoma model. They found significant uptake of delivered siRNA and a working gene silencing effect in the tumor tissue. Such treatment resulted in the attenuation of tumor growth over a 25-day period [158]. These examples are the demonstration of the great potential of gene therapy alone and in combination with carbon nanotubes in the tumor fight.

## 4. Conclusions

The unique physical properties of carbon nanotubes led, during the last decade, to an extensive exploration of such a structure as a nanoscale tool for many different applications in cancer treatment and diagnostics. The peculiar one-dimensional structure and tunable length of CNTs become an ideal platform to investigate size and shape effects in vitro, but most importantly in vivo. Unlike conventional inorganic nanoparticles, CNTs are easily functionalizable in many different ways and posses intrinsic physical properties, including resonance photoluminescence, strong NIR optical absorption and Raman scattering, which can be exploited for multiple purposes, like static and functional imaging, passive and active tracking, drug and gene vectoring and targeting. Most of these “abilities” can be joined together on a single CNT, making them a unique platform for potential multimodality cancer therapy and imaging. They can also be used in synergy with standard tumor treatment, like chemo- and radio-therapy.

Despite numerous encouraging results using CNTs in the tumor fight having been published in the past few years, much more work is still needed before they can enter the clinic. The potential long-term toxicity is still the major challenge and actual limitation to be addressed. Much has been done to solve this issue, especially with polymer coating and induced biodegradation, but still, more work is needed to obtain a very stable and non-toxic hybrid.

With these premises, they have great potential to be the tumor nano-theranostic tool of the future, that is the combination of diagnostic and therapeutic capabilities in a single nanometric agent, and may bring unknown opportunities for the future of cancer diagnosis and therapy.
